# Risk factors for nursing home admission among older adults: Analysis of basic movements and activities of daily living

**DOI:** 10.1371/journal.pone.0279312

**Published:** 2023-01-27

**Authors:** Akira Sagari, Takayuki Tabira, Michio Maruta, Koji Tanaka, Naoki Iso, Takuhiro Okabe, Gwanghee Han, Masahiro Kawagoe

**Affiliations:** 1 Department of Occupational Therapy, School of Health Sciences, Faculty of Medicine, Shinshu University, Nagano, Japan; 2 Department of Occupational Therapy, School of Health Sciences, Faculty of Medicine, Kagoshima University, Kagoshima, Japan; 3 Department of Occupational Therapy, School of Health Sciences, Faculty of Medicine, Nagasaki University, Nagasaki, Japan; 4 Graduate School of Health Sciences, Gunma University, Gunma, Japan; 5 Department of Rehabilitation, Faculty of Health Sciences, Tokyo Kasei University, Saitama, Japan; 6 Department of Occupational Therapy, School of Health Sciences at Fukuoka, International University of Health and Welfare, Fukuoka, Japan; 7 Graduate Course of Health and Social Services, Saitama Prefectural University, Saitama, Japan; University of Verona, ITALY

## Abstract

This retrospective study aimed to clarify the risk of older adults’ nursing home placement in terms of basic movements and activities of daily living (ADLs) by analyzing data from a long-term care insurance certification survey in 2016‒2018 in City A. Of the 21,520 people certified as needing care, 16,865 could be followed up until 2018. Data on sex, age, household structure, and level of care required were obtained. Those who lived at home and at nursing homes were categorized as the “Unchanged group” and the “Changed group,” respectively. Multivariate binomial logistic regression analysis was performed, with group type as the dependent variable and basic movement and ADL scores as the independent variables. For factor analysis according to care level, participants were classified into support need levels 1 and 2, care need levels 1 and 2, and care need levels 3, 4, and 5. For those categorized into support need levels 1 and 2, standing on one leg and transferring (basic movements) and urination and face cleaning (ADLs) were associated with nursing home placement. For those in care need levels 1 and 2, getting up and transferring (basic movements) and bathing, urination, face cleaning, and hair styling (ADL) were significantly associated with nursing home placement. For those in care need levels 3, 4, and 5, sitting and transferring (basic movements) and self-feeding and defecation (ADL) were significant. Occupational therapists must focus on older adults’ declining ADLs and basic movements and relay the necessary information to patients, families, and other healthcare professionals to ensure appropriate and prompt care delivery.

## Introduction

In recent years, with the rise in global life expectancy, the aging rate is increasing rapidly [1]. In Japan, older adults accounted for 28.8% of the total population in 2020 [2]. Typically, when older adults experience illnesses that limit their daily activities, they are initially provided with care at home. However, when these limitations considerably disrupt their caregivers’ lives, older adults are often admitted to nursing homes [3, 4]. Some people may want to spend the last days of their lives at home, where they have always lived. Although family members who have been caring for patients often feel guilty about placing their loved ones in nursing homes, doing so helps reduce their burden [5, 6]. Moreover, institutionalized patients may feel abandoned by their families, their sense of well-being may decline [7], dementia may progress in diagnosed patients, and depression and anxiety symptoms may appear or become more severe [8].

In addition, there are several reports of older adults experiencing abuse during their stay at nursing homes [9]. Although admission to nursing homes is not necessarily bad, geriatric researchers and caregivers have taken a keen interest in the factors associated with nursing home placement, and studies have investigated the risk of nursing home placement from various perspectives. These factors include (a) the care recipients’ sex: women are more likely to be admitted to nursing homes [10] and care recipients are more likely to enter nursing homes when their caregiver’s workload is high [11]; (b) living status: care recipients are less likely to enter nursing homes if they are being cared for by their daughters or spouses [12]; (c) family financial status: care recipients are more likely to be admitted to a nursing homes if their caregiver has an annual income of $10,000 or less [13]; (d) care recipients’ age: older care recipients are more likely to be admitted to nursing homes [14]; (e) cognitive status: care recipients are more likely to be admitted to a nursing home as their cognitive function declines [15]; (f) weight loss: care recipients are more likely to be admitted to nursing homes if they lose weight [16]; (g) behavioral and psychological symptoms of dementia: care recipients are more likely to be admitted to a nursing home if they show such symptoms of dementia [17]; and (h) basic movements and activities of daily living (ADLs) associated with older adults’ risk of admission to nursing homes [18–20].

While many international reports have described factors related to nursing home admission, the factors that contribute to admission to a nursing home in Japan have not been clarified from the perspective of basic activities and ADLs. In general, basic movement and ADL practice are emphasized in rehabilitation. Therefore, it is important for occupational/physical therapists practicing rehabilitation to know which basic movements and ADLs are factors for admission to nursing homes. Therefore, this study aimed to identify the risk factors for older adults’ admission to nursing homes based on basic movements and ADL items by analyzing long-term care insurance (LTCI) certification survey data.

## Methods

**Fig 1:**
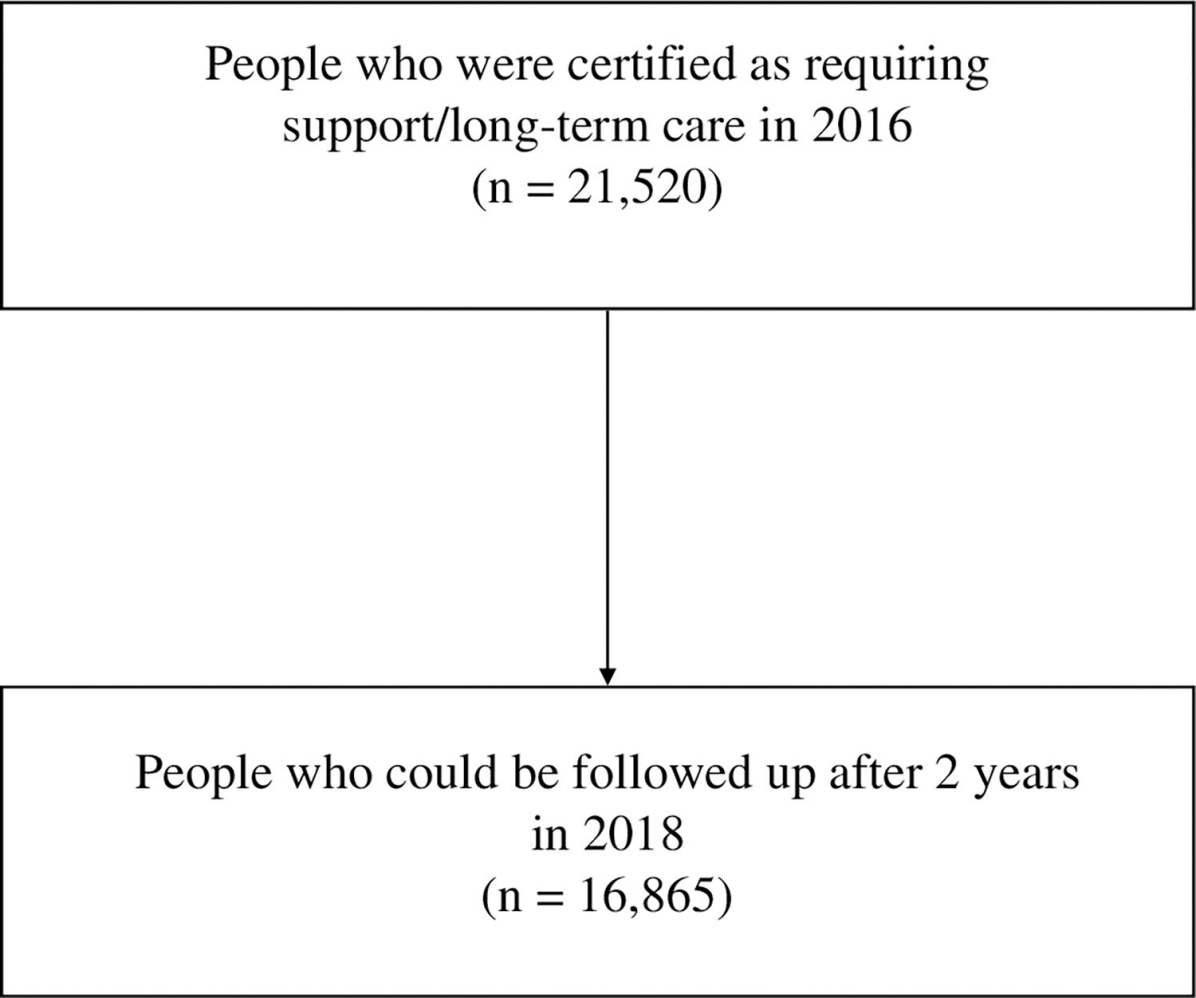
Flowchart of participant enrollment.

In this retrospective study, we used data from an LTCI certification survey conducted in City A from 2016 to 2018. As of 2016, City A’s population was 500,000, with urban development in the plains and rural habitation in the mountains and by the sea. Of these, 21,520 people were certified as needing support or care, and 16,865 could be followed up until 2018. Data on basic attributes such as sex, age, living status (alone or not alone), and level of care required were collected. Fig 1 shows a flowchart of participant enrollment.

LTCI is a system whereby society supports people and their families who need long-term care in Japan. Under this system, all residents aged 40 years and older become insured persons and pay an insurance premium. When they need long-term care or support and qualify for such care or support, they can use LTCI services by paying a portion of expenses for these services. There are seven levels of care certification: support need levels 1–2 and care need levels 1–5. The first two levels are referred to as “support need levels 1 and 2,” wherein preventive services are provided for individuals with less intensive needs. The remaining five levels, “care need levels 1–5,” provide services for individuals categorized by their severity of disability. A higher level indicates greater care need.

This study used LTCI accreditation survey data, which are assessed by trained and accredited staff and serve as material for the assessment of LTCI levels. The LTCI certification survey consists of 74 items, including 62 psychosomatic illness–related items and 12 special medical care items. In this study, data on basic movements and ADLs were analyzed.

Activities associated with basic movement such as turning over on the bed, getting up, standing on both legs, walking, standing up, and standing on one leg were assessed using a three-point scale (independent, needs watching, and needs assistance). Meanwhile, sitting and transferring were evaluated using a four-point scale (independent, needs watching, needs partial assistance, and needs full assistance).

Under ADL, bathing, self-feeding, urination, defecation, and donning and doffing upper and lower body clothes were evaluated using a four-point scale (independent, needs supervision, needs partial assistance, and needs full assistance). Meanwhile, nail-trimming, oral hygiene, face cleaning, and hairstyling were assessed using a three-point scale (independent, needs partial assistance, and needs full assistance).

In this study, we defined older adults who continued to live at home during the study period as the “home” group and those who were placed in nursing homes as the “nursing home” group. We defined nursing homes to include geriatric health services facilities, special elderly nursing homes, and medical and nursing care sanatoriums, as per standard use in Japan.

### Statistical analysis

Multivariate binomial logistic regression analyses were performed with group type (Changed/Unchanged) as the dependent variable and basic movement and ADL scores as the independent variables. Basic movement and ADL scores were calculated based on the differences in activity ratings from 2016 to 2017. For factor analysis according to care level, the participants were categorized into support need levels 1 and 2, care need levels 1 and 2, and care need levels 3–5. Basic movement activities and ADLs were analyzed separately. Sex, age, and living status were controlled for in the multivariate analysis. The variance inflation factor was used to detect multicollinearity between the explanatory variables, and no multicollinearity problem was found. Multivariate analyses were performed using the variable reduction method. The results of the analyses are presented as odds ratios (ORs) and 95% confidence intervals (95% CIs). SPSS Statistics version 27 (IBM Corp., Armonk, NY, USA) was used for all statistical analyses. The significance level was set at 5%.

Saitama Prefectural University and City A signed a memorandum on data handling according to the local government regulations for privacy policy. Additionally, the Ethics Committee of Saitama Prefectural University approved the procedure of this study with an anonymized database (No. 20019). No written or verbal informed consent was obtained, as the study involves the use of big data in compliance with the Personal Data Protection Ordinance of City A.

## Results

Table 1 shows the individual baseline characteristics of the home group (n = 15,928) and nursing home group (n = 937).

**Table 1:** Traceable participants’ baseline characteristics in 2016 categorized by group (N = 16,865).

Items	Home	Nursing home
N	15,928	937
Sex		
Male	4,933	271
Female	10,995	666
Age, years (mean ± SD)	82.53 ± 7.23	83.77 ± 7.34
	Support needs	
Level 1	2,498	26
Level 2	3,725	75
	Care needs	
Level 1	3,539	194
Level 2	3,042	231
Level 3	1,637	163
Level 4	902	157
Level 5	585	676
	Living status	
Alone	7,067	445
Not alone	8,861	492

*Note*. Definition of support and care needs levels. Support needs level 1: The person can rise, walk, and perform the most essential daily life activities by themselves. However, they need some support for task-based activities in daily life, including cooking, shopping, and taking oral medicine. Support needs level 2: The person’s ability to handle task-based activities in daily life is slightly lower than that of individuals in the support needs level 1 category. They need more support. Care needs level 1: The person faces difficulty in performing essential daily life activities by themselves. The person’s ability to handle task-based activities in daily life is lower than that of individuals in the support needs level 2 category. Care needs level 2: The person is in a similar state as described in care needs level 1 but requires more care to perform essential daily life activities. Care needs level 3: Compared with individuals from care needs level 2, the person’s abilities to perform essential daily life activities and task-based activities are significantly lower. They require almost constant care. Care needs level 4: This person is in a state like that detailed under care needs level 3, but their ability to act is lower. They face difficulty living without constant care. Care needs level 5: The person’s ability to act is even lower than that of individuals in the care needs level 4 category. They require almost constant care to live. Outcomes: a) unchanged group: individuals continued to live in their homes; b) changed group: individuals moved to a nursing home.

Table 2 shows the results of the analysis of basic movement changes in older adults with different levels of LTCI certification two years after being placed in nursing homes. For those categorized into support need levels 1 and 2, limitations in/requiring assistance for standing on one leg (OR 2.188, 95% CI 1.223–3.915, P = 0.008) and transferring (OR 2.666, 95% CI 2.000–3.554, P < 0.001) were associated with nursing home placement. Limitations in/requiring assistance for getting up (OR 1.509, 95% CI 1.080–2.108, P = 0.016) and transferring (OR 1.620, 95% CI 1.414–1.855, P < 0.001) were statistically significantly associated with nursing home placement in those with care need level 1 or 2. For those categorized into care need levels 3, 4, or 5, limitations in/requiring assistance for sitting up (OR 1.279, 95% CI 1.048–1.562, P = 0.015) and transferring (OR 1.188, 95% CI 1.046–1.349, P = 0.008) were statistically significant factors in nursing home placement.

**Table 2:** Risk factors for nursing home placement related to basic movements.

Items/Activities	Support needs 1 and 2				Care needs 1 and 2				Care needs 3, 4, and 5			
	OR	95% CI	p	R^2^	OR	95% CI	p	R^2^	OR	95% CI	p	R^2^
Getting up				0.021	1.509	1.080–2.108	0.016	0.023				0.008
Sitting									1.279	1.048–1.562	0.015	
Standing on one leg	2.188	1.223–3.915	0.008									
Transferring	2.666	2.000–3.554	< .001		1.620	1.414–1.855	< .001		1.188	1.046–1.349	0.008	

*Note*. OR = odds ratio, CI = confidence interval, R^2^ = coefficient of determination

Logistic regression analysis (variable reduction method).

Sex, age, and living status were controlled for in this analysis.

Table 3 shows changes in ADLs in older adults with different levels of LTCI certification two years after being placed in nursing homes. For those at support need levels 1 and 2, limitations in/requiring assistance for urination (OR 1.741, 95% CI 1.266–2.394, P < 0.001) and face cleaning (OR 1.902, 95% CI 1.011–3.579, P = 0.046) were statistically significantly associated with nursing home placement. For those categorized into care need level 1 or 2, limitations in/requiring assistance for bathing (OR 1.203, 95% CI 1.021–1.419, P = 0.028), urination (OR 1.257, 95% CI 1.115–1.417, P < 0.001), face cleaning (OR 1.292, 95% CI 1.028–1.624, P = 0.028), and styling hair (OR 1285, 95% CI 1.070–1.543, P = 0.007) were significant factors, whereas for those at care need level 3, 4, or 5, limitations in/requiring assistance for self-feeding (OR 1.168, 95% CI 1.023–1.334, P = 0.022) and defecation (OR 1.207, 95% CI 1.046–1.394, P = 0.010) were statistically significant factors in nursing home placement.

**Table 3:** Risk factors for nursing home placement related to activities of daily living.

Items/Activities	Support needs 1 and 2				Care needs 1 and 2				Care needs 3, 4, and 5			
	OR	95% CI	*p*	R^2^	OR	95% CI	*p*	R^2^	OR	95% CI	*p*	R^2^
Bathing				0.22	1.203	1.021–1.419	0.028	0.023				0.006
Self-feeding									1.168	1.023–1.334	0.022	
Urination	1.741	1.266–2.394	< .001		1.257	1.115–1.417	< .001					
Defecation									1.207	1.046–1.394	0.010	
Face cleaning	1.902	1.011–3.579	0.046		1.292	1.028–1.624	0.028					
Styling hair					1.285	1.070–1.543	0.007					

*Note*. OR = odds ratio, CI = confidence interval, R^2^ = coefficient of determination

Logistic regression analysis (variable reduction method).

Sex, age, and living status were controlled for in this analysis.

## Discussion

The risk factors for nursing home placement among older adults are a widespread concern in the field of geriatrics. Although research has examined various factors related to older adults’ nursing home placement in terms of basic movements and ADLs [18–20], to our knowledge, However, the factors that contribute to admission to a nursing home in Japan have not been clarified in detail from the perspective of basic activities and ADLs.

Regarding basic movements, decreased abilities to transfer and stand on one leg were significant factors in nursing home placement for those at support need levels 1 and 2. For those at care need levels 1 and 2, decreased abilities to transfer and get up were associated with nursing home placement. Meanwhile, in the case of those at care need levels 3, 4, and 5, decreased abilities to transfer and sit were significant factors. Assisting in transferring can cause back pain in caregivers, and reduced transferring ability significantly increases caregiver burden [21–23]. Various welfare interventions have been developed to reduce caregiver burden for transferring [24]. In addition, many daily life situations, such as toileting, bathing, and going out, involve transferring, which can affect nursing care in general. Therefore, reduced ability to transfer was determined as a risk factor for nursing home placement regardless of the care need level.

This study also showed that for those at support need levels 1 and 2, reduced ability to stand on one leg is a factor in nursing home placement. Frail older adults have a lower ability to balance and a higher risk of falling [25, 26]. Furthermore, impaired balance is a predictor of ADL deterioration [27], and older adults with impaired balance require constant attention to prevent falls, which further increases caregiver burden. Therefore, the decline in standing on one leg may be a factor in admission to an institution for the older adults.

In those at care need levels 1 and 2, the decline in the ability to get up, in addition to transferring, was associated with nursing home placement. In particular, caregiver burden increases as older adults’ ability to get up decreases [28]. Therefore, caregivers of family members often have no choice but to turn to nursing homes to provide care for older adults.

In those at care need levels 3, 4, and 5, a decline in sitting ability, in addition to transferring, was the most important factor. The ability to hold a sitting position was directly related to the amount of assistance required for all ADL items. For example, caregivers must be present at all times when patients with sitting difficulties use fixed or portable toilets; this increases the burden on both parties [29]. In addition, an unstable sitting ability makes it difficult for patients to don and doff their shoes and clothes. Thus, we suggest that poor sitting ability is a risk factor for nursing home placement in older adults at care need levels 3, 4, and 5.

In terms of ADLs, declines in urination and face cleaning abilities were risk factors for nursing home placement in older adults at support need levels 1 and 2. Urination care significantly increases caregiver burden [30] and is an ADL item that caregivers may sincerely wish for care recipients’ independence. Face cleaning comprises a series of actions, such as turning on the faucet, washing the face, and wiping the face with a towel, and these require the coordination of both upper limbs. Furthermore, face cleaning function requires the ability to balance and maintain posture. Although assisting older adults with face cleaning is not considered a significant burden, the difficulty in coordinating the movements of both upper limbs as well as reduced balance may spill over to other ADLs, thereby becoming a risk factor for nursing home placement.

For those at care need levels 1 and 2, in addition to urination and face cleaning, bathing and styling hair were associated with nursing home placement. Styling hair requires skillful handling of the comb with one hand. When combing hair, it is necessary to precisely manipulate the back of the head with a comb; this cannot be performed well by those with muscle weakness and a limited range of motion. Washing involves cleaning the entire body with a soap or hand towel. In particular, high flexibility and manipulation of the upper limbs are essential when washing the back and lower limbs. However, washing requires the assistance of a caregiver as it is performed in a wet, slippery place, entailing a higher risk of falls [31]. Thus, washing, too, places a heavy burden on caregivers and calls for a high level of caregiving skills. In Japan, the use of home-visit bathing care services increases as care recipients’ care needs increase [32]. Therefore, a decline in older adults’ washing ability is associated with nursing home placement.

For those at care need levels 3, 4, and 5, declines in defecation and self-feeding abilities were associated with nursing home placement. Similar to urination, defecation is an ADL for which the care recipient prefers independence. For older adults at care need levels 3, 4, and 5, defecation is more likely to occur in bed. Joint contractures [33] and frailty [34] make defecation care more difficult. In addition, incontinence-associated dermatitis increases the risk of pressure ulcers [35]. Therefore, it is imperative that family caregivers provide adequate assistance for defection to those at care need levels 3, 4 and 5. Furthermore, self-feeding requires various types of assistance, such as serving food, arranging plates, and using spoons and chopsticks. In addition, many older adults at care need levels 3, 4, and 5 have dysphagia, and appropriate care practices are required to prevent aspiration pneumonia. In addition, since self-feeding is usually performed thrice a day, it places a heavy burden on caregivers. It is also one of the most important ADLs that can be easily maintained [36]. Therefore, a decline in self-feeding ability may reflect the disruption of all other ADLs. It is reasonable to suggest that a decline in self-feeding ability is associated with nursing home placement among older adults at care need levels 3, 4, and 5.

Strategies to reduce the risk of nursing home placement include caregiver support [37], respite care day programs [38], case management [39], and home visits by healthcare professionals [40]. Although these strategies have been noted for their potential to reduce the risk of nursing home admission, extant studies do not provide sufficient evidence, and further research is needed [41].

### Limitations

In this study, we focused on basic movements and ADL. However, we could not analyze data that were not included as covariates, such as the income, marital status, and education of care recipients as well as caregivers. Therefore, the results should be interpreted with caution. Furthermore, because our analysis was based on data from an LTCI certification survey, we could not obtain direct information from caregivers regarding their degree of caregiver burden. Notably, these data are for City A, and may not reflect LTCI data for Japan as a whole. Following the implementation of the Care Insurance system, the number of residents in facilities not officially categorized as "institutional care" has increased greatly: for example, those resident in paid nursing homes, group homes and housing with services. The elderly using these facilities are not assigned to the "nursing homes" category within this study. In addition, to be admitted to the special elderly nursing homes, there is a delay due to waiting lists. Therefore, caution should be exercised when interpreting the results of this study.

## Conclusion

This study examined the risk factors related to basic movements and ADL that contribute to older adults’ admission into nursing homes by categorizing them into three LTCI care level groups. To reduce the risk of nursing home placement, it is necessary to monitor and weigh the risk items/activities identified in this study according to each care need level and to train caregivers (family members living with older adults) on providing effective care with minimal burden. Occupational and physical therapists must pay close attention to older adults’ declining movement abilities and ADL based on their care need level and share this information with patients, families, and health care professionals to facilitate appropriate and timely interventions. Furthermore, occupational and physical therapists must provide a safe and secure environment where patients can perform various ADLs, including transferring. They should refer to the results of this study and support care recipients in maintaining their abilities.
